# Complete remission of heavily treated ovarian clear cell carcinoma with *ARID1A* mutations after pembrolizumab and bevacizumab combination therapy: a case report

**DOI:** 10.1186/s13048-020-00751-3

**Published:** 2020-12-08

**Authors:** Yu-Chien Lin, Kuo-Chang Wen, Pi-Lin Sung, Yu-Ting Chou, Phui-Ly Liew, Lin-Yu Chen, Rui-Lan Huang, Hung-Cheng Lai, Lu-Te Chang

**Affiliations:** 1 Department of Obstetrics and Gynecology, Shuang Ho Hospital, Taipei Medical University, New Taipei City, 23561 Taiwan; 2 Department of Obstetrics and Gynecology, School of Medicine, College of Medicine, Taipei Medical University, Taipei, Taiwan; 3 Department of Pathology, Shuang Ho Hospital, Taipei Medical University, New Taipei, Taiwan; 4 Department of Pathology, School of Medicine, College of Medicine, Taipei Medical University, Taipei, Taiwan; 5 Translational Epigenetic Center, Shuang Ho Hospital, Taipei Medical University, New Taipei City, Taiwan

**Keywords:** Angiogenesis inhibitor, *ARID1A*, Checkpoint inhibitor, Ovarian clear cell carcinoma

## Abstract

**Background:**

Patients with ovarian clear cell carcinoma (OCCC) have a poor prognosis because they show low sensitivity to platinum-based chemotherapy. New treatments for refractory OCCC are urgently needed.

**Case presentation:**

We present a patient with refractory OCCC in whom conventional chemotherapy failed. Cachexia was induced by the disseminating recurrent tumors. Tumor tissue staining and genomic analysis revealed PD-L1 negativity, a low tumor burden, stable microsatellite instability, and two mutations in *ARID1A*. The patient was administered pembrolizumab combined with bevacizumab triweekly. Her serum CA-125 level decreased dramatically after the first cycle. A computerized tomography scan showed marked regression of the recurrent masses after 3 cycles, and the patient reached complete remission after 9 cycles. She showed good recovery from cachexia. We observed no marked side effects except for mild polyarthritis of the small joints.

**Conclusions:**

The therapeutic effect of checkpoint inhibitors combined with angiogenesis inhibitors is very promising in our patient with OCCC. Further clinical trials of tumors including *ARID1A* mutations are warranted.

## Background

Epithelial ovarian cancer (EOC) is one of the most lethal malignancies in the female reproductive system. Various subtypes of EOC exhibit histological and genomic heterogeneity [10]. Among these different subtypes, ovarian clear cell carcinoma (OCCC) remains challenging to treat [5]. Although optimal debulking surgery followed by platinum-based chemotherapy is the standard therapy for EOC, OCCC can easily become resistant to these conventional treatments [3]. Some studies have suggested that the poor prognosis of OCCC is related to distinct molecular characteristics [13]. Molecular variations, such as *ARID1A* mutations and overexpression of vascular endothelial growth factor (VEGF), annexin A4, and mammalian target of rapamycin (mTOR), have been reported in OCCC [5]. A precision medicine approach that targets these unique features may be a new direction for OCCC treatment.

We present a patient with refractory OCCC who was successfully treated with a combination of an immune checkpoint inhibitor, pembrolizumab, and the angiogenesis inhibitor bevacizumab. This case report was approved by the institutional review board of Shuang Ho Hospital (N201909048).

## Case presentation

A 42-year-old Asian woman, gravida 0, para 0, underwent laparoscopic cystectomy for a suspected ovarian chocolate cyst at Kaiser Hospital in southern California, USA, in March 2014. Pathology revealed clear cell carcinoma. An optimal debulking operation was subsequently performed, and the patient was found to have FIGO stage II disease. She was administered adjuvant chemotherapy with paclitaxel and carboplatin for 7 cycles. However, an increasing serum CA-125 level and recurrent pelvic tumors were noted in January 2016. She underwent a secondary debulking operation, followed by administration of adjuvant chemotherapy using carboplatin and gemcitabine. However, secondary recurrence deep in the pelvic cavity close to the sigmoid colon, rectum, and bladder was found in September 2017. Her recurrence progressed despite the administration of salvage chemotherapy, including liposomal doxorubicin and topotecan. In February 2019, she presented to a medical center in Taiwan and underwent a third debulking surgery including resection of the sigmoid colon, rectum, and bladder, followed by small bowel bypass, T-colostomy and bilateral percutaneous nephrostomy. Tumor recurrence occurred, with two major masses observed in the pelvis and abdomen soon after surgery. Palliative treatment was suggested because she was refractory to cancer treatment. Immune cell therapy with unknown immunological cells was attempted at a clinic but was ineffective.

**Fig. 1:**
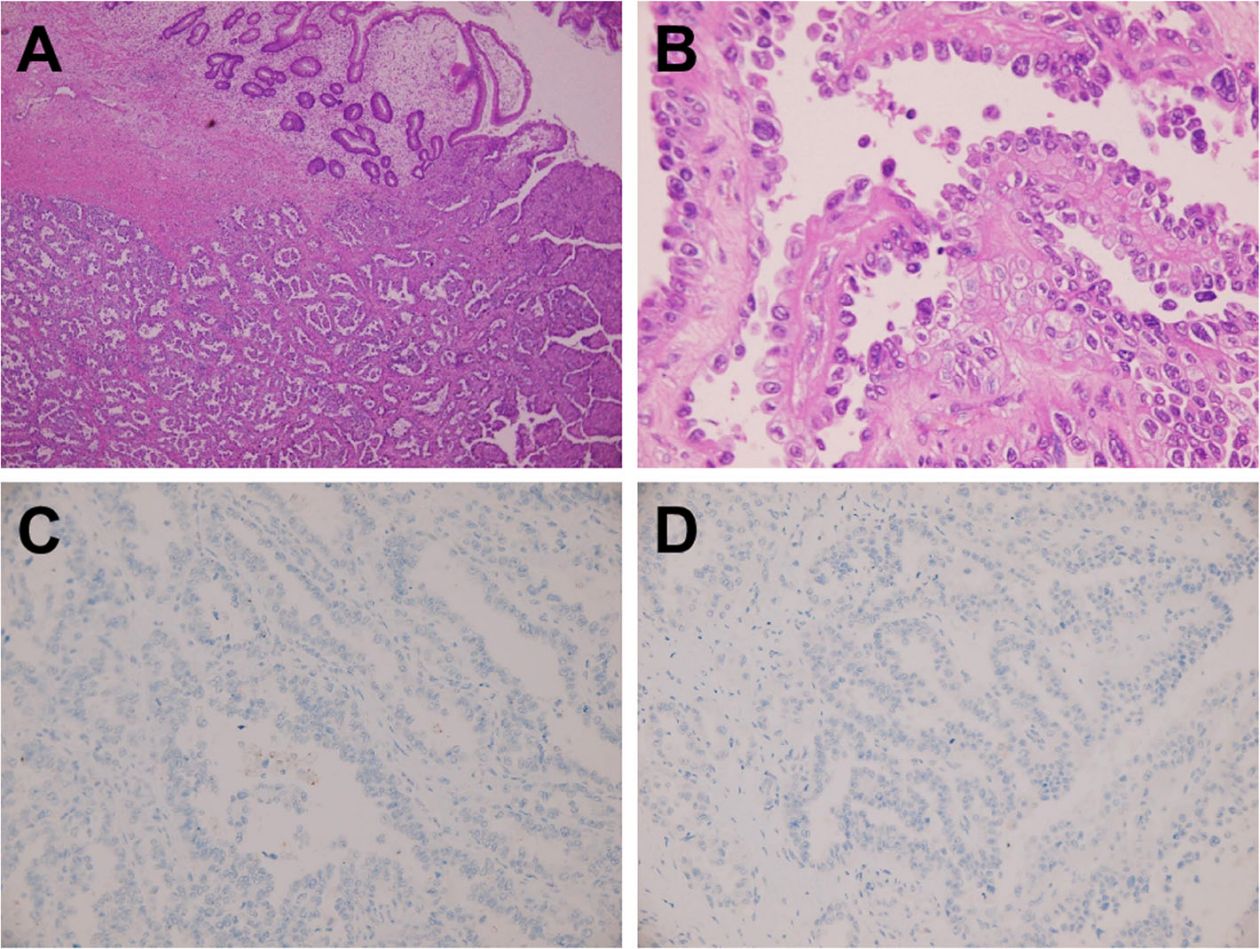
Immunohistochemistry (IHC) of a recurrent tumor (China Medical University Hospital). **a** Representative histological features (H&E, 100×). **b** Representative histological features (H&E, 400×). **c** Corresponding images of PD-L1 staining (IHC 22C3 pharmDx Assay, Agilent/Dako, 400×). **d** Corresponding images of PD-L1 staining (IHC 28–8 pharmDx Assay, Agilent/Dako, 400×)

**Fig. 2:**
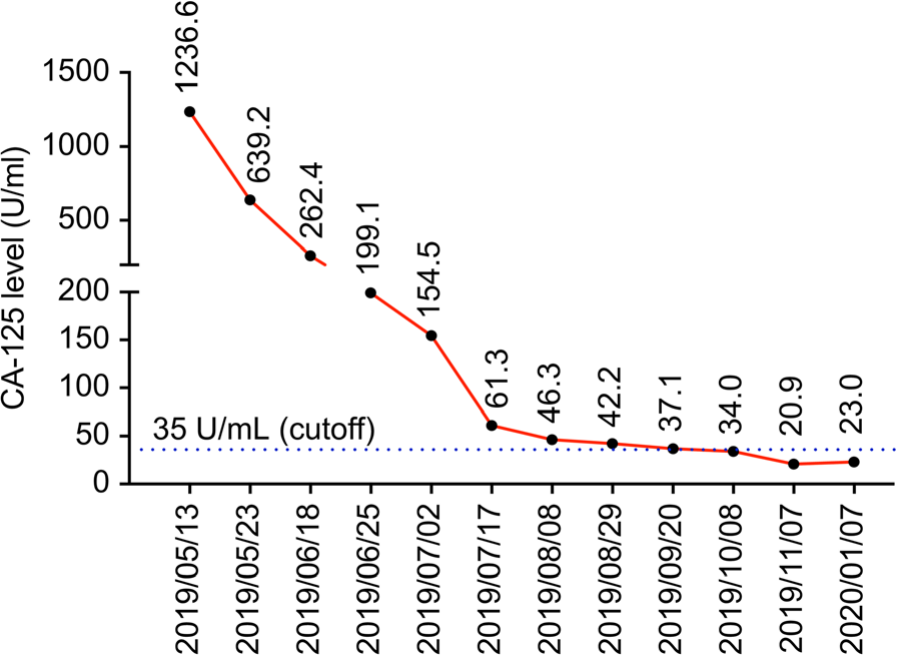
Serum CA125 levels according to the dual combination therapy timeline. The serum CA125 level decreased significantly during 9 cycles of dual therapy (dates of therapy are shown as red triangles: 05/13/2019, 06/04/2019, 06/25/2019, 07/17/2019, 08/08/2019, 08/29/2019, 09/20/2019, 10/11/2019, and 11/07/2019)

**Fig. 3:**
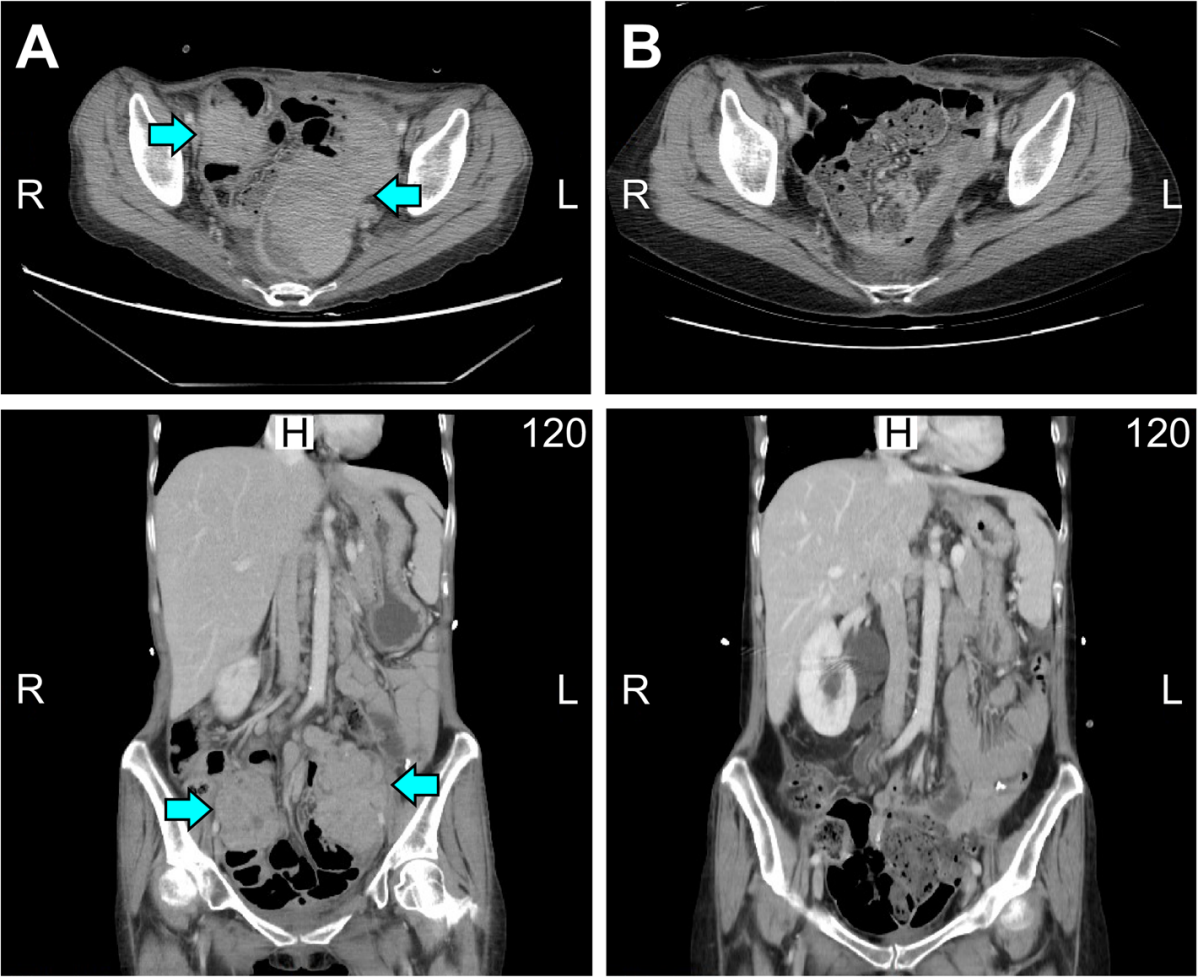
CT scan before and after the dual combination therapy. **a** Recurrent pelvic tumors (yellow arrow) were noticed in May 2019 (upper panel: axial view; lower panel: sagittal view). **b** Significant regression of recurrent pelvic tumors was found in November 2019 (upper panel: axial view; lower panel: sagittal view)

**Fig. 4:**
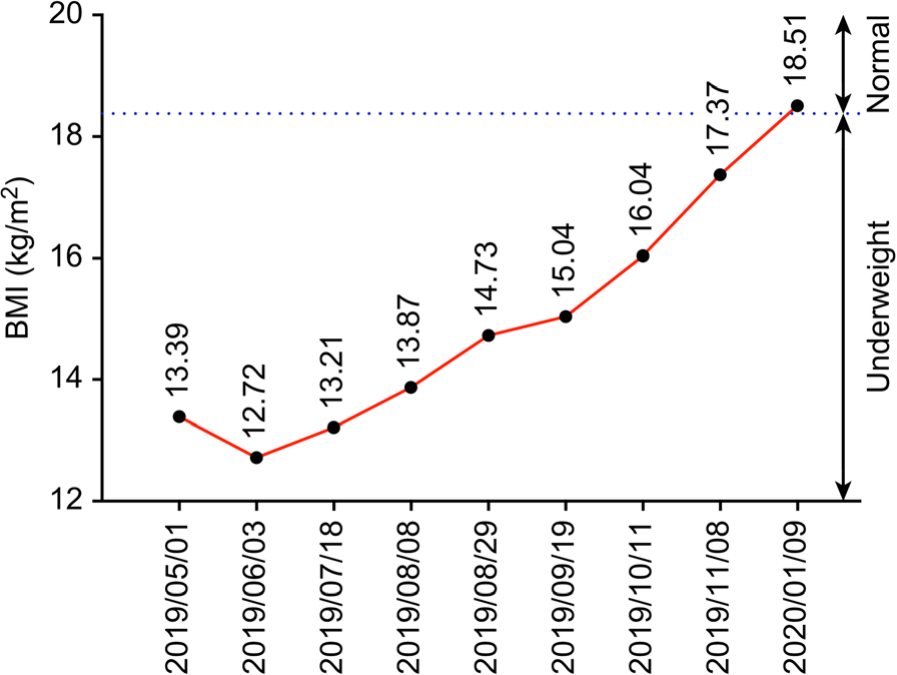
BMI according to the dual combination therapy timeline. The patient’s BMI value grossly increased during 9 cycles of dual therapy (dates of therapy are shown as red triangles: 05/13/2019, 06/04/2019, 06/25/2019, 07/17/2019, 08/08/2019, 08/29/2019, 09/20/2019, 10/11/2019, and 11/07/2019)

She presented to our hospital with a high CA125 level, a pelvic mass with resultant vaginal bleeding, and severe cachexia in April 2019. Based on her history, genetic analysis of more than 300 genes was performed (Foundation Medicine, FoundationOne CDx) and revealed a stable microsatellite status, low mutation burden, and two mutations in *ARID1A* (Table 1). Immunohistochemical staining of PD-L1 was negative (Fig. 1; positive control staining was performed in tonsil tissue, Supplementary Figure 1). After discussion, the patient and her family agreed to treatment with a checkpoint inhibitor combined with bevacizumab, with the understanding that the checkpoint inhibitor alone would not effectively treat EOC based on previous clinical trials. The patient was administered pembrolizumab (200 mg) combined with bevacizumab (15 mg/kg; 400 mg) every 3 weeks. Her serum CA-125 level dramatically decreased from 1236.6 to 639.2 U/mL after 1 cycle of treatment; her CA-125 level reached the normal range (35 U/mL) after 7 cycles (Fig. 2). Computerized tomography (CT) scanning also showed significant regression of recurrent masses and a partial response at 3 months after beginning treatment. The patient’s disease achieved complete remission after 9 cycles (Fig. 3). She recovered from cachexia to a normal body mass index (Fig. 4), as evidenced by an increase in subcutaneous fat and muscle in axial view CT images, as shown in Fig. 2. There were no adverse effects, such as hypertension, pneumonitis, colitis, or hepatitis, except for small joint arthritis in both hands in later cycles. As of the time of preparation of this manuscript, the patient has remained disease-free.

**Table 1:** Genomic analysis (FoundationOne CDx) of recurrent tumors in the colon (China Medical University Hospital)

Finding	Result
Biomarkers	
Microsatellite status (MS)	Stable
Tumor mutational burden (TMB)	Low
Genomic data	
*ARID1A*	M1154fs*7, Y788*
*ERBB2*	Amplification
*APC*	G2400*
*GABRA6*	K110T
*TERT* promoter	124C > 7
*TP53*	R280K

## Discussion and conclusions

Pembrolizumab (trade name Keytruda) is a humanized antibody that targets programmed cell death protein 1 (PD-1) on lymphocytes. In 2014, the US Food and Drug Administration approved pembrolizumab for treatment of melanoma, non-small cell lung cancer, head and neck squamous cell cancer, Hodgkin lymphoma, urothelial carcinoma, stomach cancer, solid tumors with mismatch repair deficiency or microsatellite instability, and recurrent or metastatic cervical cancer expressing PD-L1, but the drug was not approved for ovarian cancer [11]. The phase II KEYNOTE-100 study (ClinicalTrials.gov identifier: NCT02674061) recruited 376 patients with recurrent ovarian cancer for pembrolizumab monotherapy (intravenous, triweekly, 200 mg for 35 cycles). The objective response rate was only 8.0% (1.9% complete response and 6.1% partial response). The median progression-free survival was 2.1 months (95% CI, 2.1–4.1) [6]. According to the KEYNOTE-100 study, PD-L1 expression was considered a predictive biomarker for the therapeutic response of pembrolizumab. However, our patient was negative for PD-L1 expression in the tumor. Her unexpected excellent response supports the need to identify other theranostic biomarkers for checkpoint inhibitors [1]. Because OCCC is prevalent in Asia [7], a subgroup analysis of 21 Japanese patients with OCCC in the KEYNOTE-100 trial revealed a better objective response rate of these patients (19.0, 95% CI: 5.4–41.9). The hypothesis and the reasons why OCCC showed a better response to immune checkpoint inhibitors require further investigation.

Mutations in *ARID1A* may be predictive for immune checkpoint inhibitors in OCCC [13]. *ARID1A* (AT-rich interactive domain-containing protein 1A), an important subunit of the SWI/SNF (SWItch/Sucrose NonFermentable) chromatin remodeling complex, can alter the positions of nucleosomes along DNA [14]. The mammalian SWI/SNF complex is considered a tumor suppressor in several human malignancies and plays an important role in endometriosis-associated ovarian cancer [14]. *ARID1A* can recruit *MSH2* to chromatin during DNA replication and promote mismatch repair, and *ARID1A* inactivation compromises mismatch repair and increases mutagenesis and neoantigen levels [12]. In a syngeneic mouse model, an *ARID1A*-deficient ovarian cancer tumor displayed an increased mutation load, increased PD-L1 expression, and elevated numbers of tumor-infiltrating lymphocytes because of neoantigen production. Treatment with an anti-PD-L1 antibody reduced the tumor burden and prolonged the survival of mice bearing *ARID1A*-deficient but not *ARID1A* wild-type ovarian tumors. In addition to increased PD-L1, *ARID1A* may regulate other factors associated with oncogenic pathways such as *PIK3CA*-activating mutations, DNA methylation, or VEGF [14]. The levels of these signatures and markers should be determined after therapy using a liquid biopsy with genetic/epigenetic analysis in the future. Our patient had two frame shifts in *ARID1A* (p.M1154fs*7 and p.Y788*). In a clinical cohort report, progression-free survival after immune checkpoint blockade therapy was significantly longer in patients with *ARID1A*-altered tumors (*n* = 46) than in patients with wild-type *ARID1A* (*n* = 329) (*p* = 0.006) [8]. In multivariate analyses, *ARID1A* alterations could predict longer progression-free survival after checkpoint blockade (95% CI 0.39 to 0.94, *p* = 0.02), independent of microsatellite instability or the mutational burden. These reports demonstrate that the functional loss of *ARID1A* may act in conjunction with immune checkpoint blockade therapy.

Bevacizumab (trade name Avastin) is an angiogenesis inhibitor that blocks vascular endothelial growth factor A [2]. In recent years, bevacizumab has been added to chemotherapy as a “maintenance” strategy in front-line, recurrent cisplatin-sensitive, or cisplatin-resistant settings of ovarian cancer [4]. Two single-agent bevacizumab phase II trials (GOG-0170D and Genentech AVF 2949 g) revealed response rates of only 16–21% in recurrent ovarian cancer. Later phase III trials (GOG-0218, OCEANS, AURELIA, ICON7) suggested that bevacizumab is effective in combination with chemotherapy. An open-label phase II clinical trial on a three-factor combination (pembrolizumab, 200 mg, combined with bevacizumab, 15 mg/kg every 3 weeks, and oral cyclophosphamide, 50 mg every day) in 40 patients with platinum-sensitive or platinum-resistant recurrent epithelial ovarian, fallopian tube, or primary peritoneal cancer was presented at the annual meeting of the 2019 Society of Gynecologic Oncology [15]. The scientific rationale for this three-drug treatment is that lymphocyte trafficking is improved and that dendritic cell function is restored using bevacizumab. Additionally, circulating regulatory T cells become depleted, while the cytotoxic potential of T cells and natural killer cells is simultaneously restored. These effects help increase the efficacy of pembrolizumab. The 6-month progression-free survival rate was 70%, which included 15 patients who achieved a partial response and 13 who had stable disease. The investigators described the combination as “well-tolerated”. Another multicenter, randomized phase 2 study (Clinical Trial NCT02337491) of recurrent glioblastoma patients treated with pembrolizumab with (cohort A) and without bevacizumab (cohort B) revealed a median follow-up of 25.3 months and median progression-free survival of 4.1 months in cohort A and 1.4 months in cohort B. The proportion of 6-month progression-free survival was 26.0% in cohort A (95% CI, 16.3–41.5) and 6.7% in cohort B (95% CI, 1.8–25.4), suggesting an advantage of the combination of pembrolizumab and bevacizumab [9]. Both trials demonstrated tolerable side effect profiles. The current patient was not administered oral cyclophosphamide because of concerns regarding side effects from conventional chemotherapy. She did not have hypertension or other common side effects, such as pneumonitis, colitis, or hepatitis, but had only mild polyarthritis in both hands.

In conclusion, OCCC is a subtype of EOC that is prevalent in Asia and has a poor prognosis. Combination treatment with pembrolizumab and bevacizumab may be considered in OCCC, particularly with *ARID1A* mutations. Many clinical trials (NCT03596281 and NCT04361370) to determine the efficacy of this combination therapy for ovarian cancer are on the way. Since our manuscript was submitted, a phase 2 nonrandomized clinical trial (NCT02853318 study collaborated by National Cancer Institute) testing pembrolizumab combined with bevacizumab and oral metronomic cyclophosphamide in the treatment of recurrent ovarian cancer was published [16]. While this study only included four patients diagnosed with OCCC (2 pure and 2 mixed subtypes), it is not clear how they fared in the trial. The present report may provide insight into the design of further trials that test the combination of pembrolizumab and bevacizumab for OCCC.

## Supplementary Information

**Additional file 1: Supplementary Figure 1**. Immunohistochemistry (IHC) of the PD-L1 control. (a) Positive control of PD-L1 staining (Dako 22C3, tonsil). (b) Positive control of PD-L1 staining (Dako 28–8, tonsil)

## Data Availability

The data obtained during the current study are available from the corresponding author on reasonable request.

## Abbreviations

EOC: Epithelial ovarian cancer
OCCC: Ovarian clear cell carcinoma
VEGF: Vascular endothelial growth factor
mTOR: Mammalian target of rapamycin
CT: Computerized tomography
