# Supplementary figures and images for: Complete remission of heavily treated ovarian clear cell carcinoma with ARID1A mutations after pembrolizumab and bevacizumab combination therapy: a case report
Source: J Ovarian Res. 2020 Dec 8;13:143. doi: 10.1186/s13048-020-00751-3 (PMC7725117; doi:10.1186/s13048-020-00751-3)

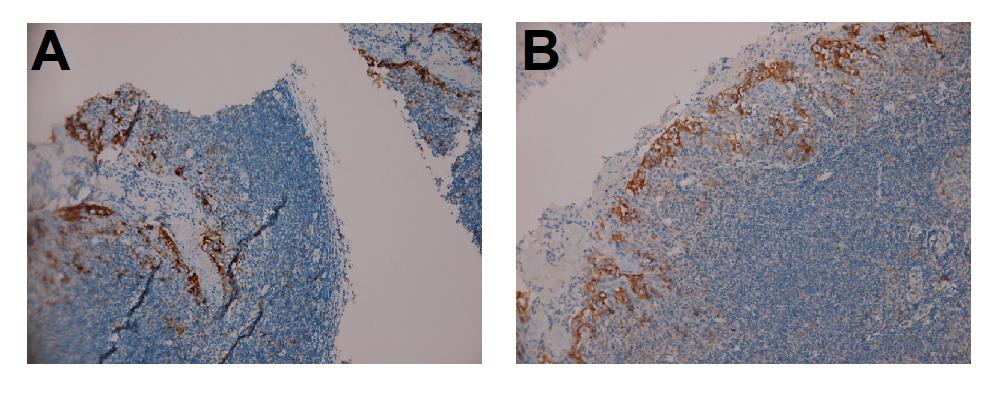

Supplement: Supplementary file 1 — Additional file 1: Supplementary Figure 1. Immunohistochemistry (IHC) of the PD-L1 control. (a) Positive control of PD-L1 staining (Dako 22C3, tonsil). (b) Positive control of PD-L1 staining (Dako 28–8, tonsil) [file 13048_2020_751_MOESM1_ESM.tif]
